# Leveling the curve of Spee using different sized archwires: a randomized clinical trial of blood flow changes

**DOI:** 10.1007/s00784-023-04894-7

**Published:** 2023-02-11

**Authors:** Raidan Ba-Hattab, Elham S. Abu Alhaija, Yousef H. Nasrawi, Nessrin Taha, Hasan Daher, Saba Daher

**Affiliations:** 1 College of Dental Medicine, QU Health, Qatar University, P.O. Box 2713, Doha, Qatar; 2 Amman, Jordan; 3 Division of Endodontics, Department of Conservative Dentistry, Faculty of Dentistry, Jordan University of Science and Technology, P.O. Box 3030, Irbid, Jordan; 4 College of Medicine, Jordan University of Science and Technology, P.O. Box 3030, Irbid, Jordan

**Keywords:** Randomized clinical trial, Curve of Spee leveling, Archwires, Blood flow, Laser Doppler flowmeter

## Abstract

**Objectives:**

To compare blood flow (BF) changes of teeth subjected to orthodontic forces during curve of Spee (COS) leveling using different archwires (AW).

**Material and methods:**

Thirty subjects with COS > 5 mm were randomly assigned (1:1:1) into three groups based on the AW used: group 1: 0.017 × 0.025-inch stainless-steel (SS)AW, group 2: 0.019 × 0.025-inch SSAW, and group 3: 0.021 × 0.025-inch β-titanium (TMA)AW. In the 3 groups, a 5 mm-depth reverse COS was placed in the AWs. A laser Doppler flowmeter was used to measure BF at different time intervals (T0–T4).

**Results:**

In the 3 AWs group, BF of all measured teeth was reduced 20 min after force application. Afterwards, the BF values started to increase until the baseline values were almost restored within 1 week. Differences in BF changes between the extrusion and intrusion subgroups were observed within groups 1 and 3 during the first 20 min of force application (*P* < 0.05). Similar BF changes were recorded using the 3 different AWs. BF changes were associated with tooth type and the amount of COS depth change.

**Conclusions:**

During CoS leveling, similar BF changes were recorded using the 3 different AWs. Tooth type and the amount of COS depth change were associated with BF changes within the first 20 min of force application. Greater BF reduction was found in premolars compared to incisors during the first 20 min of AW placement.

**Clinical relevance:**

It is important to select a type of applied forces that minimally affect the BF. Intrusive forces appeared to have lower negative effects on the BF of teeth during COS leveling.

**Trial registration:**

ClinicalTrial.gov (# NCT04549948).

## Introduction

The curve of Spee (COS) refers to the upward progression of the teeth curvature from the incisors through the premolars and molars. A deep COS is usually associated with an increased anterior overbite. During orthodontic treatment, COS is leveled by bringing the incisal edges of the anterior teeth and the buccal cusps of the posterior teeth into a horizontal plane level [1].

The COS correction is usually achieved by posterior teeth extrusion and lower incisor intrusion [2–4]. A randomized clinical trial conducted by Nasrawi et al. [5] to measure the vertical movement for the lower incisors and lower molars associated with leveling excessive COS using 3 different archwire (AW) sizes concluded that leveling the COS was achieved in all groups by incisor intrusion, molar extrusion, and incisor proclination on different levels.

The application of orthodontic forces to teeth has been reported to induce molecular changes in the cells of the periodontal ligament, alveolar bone, and the pulp–dentine complex [6, 7].

The application of a contentious force to the teeth not only results in an inflammatory reaction within the periodontium but also has a significant effect on pulpal neural responsiveness, possibly for the entire duration of the orthodontic treatment [8]. Several methods have been suggested to investigate the pulpal response to an applied force. Histological observation [6, 7], fluorescent microsphere injection, and measurement of pulp tissue respiration rate may only be applied following tooth extraction. Laser Doppler flowmetry (LDF) is a noninvasive method that can be used to obtain repeated measurements of blood flow (BF) without causing tissue damage [9]. The blood flow signals from intact teeth measured by the LDF contain information of alteration in the BF. Therefore, the technique has been used to evaluate alterations in the BF during orthodontic movements in humans [10].

Most alterations in the BF that result from orthodontic treatment are reversible unless the pulp has been previously irritated by caries, restorations, or trauma [11]. Rarely, pulpal side effects may occur, including altered pulpal respiration rate, internal root resorption, pulp obliteration, and pulpal necrosis [12]. The characteristics of the applied orthodontic forces, such as the type, magnitude, force duration, and distribution, could contribute to blood flow disturbance [6, 12–14] and make the alteration reversible or irreversible. It is unanimously accepted that dental pulp changes are directly proportional to the applied forces; therefore, in adults it has to be between 50 and 100 cN [15]. High forces increase the risk of radicular resorption by raising the hyalinization of the periodontal tissue and inducing very sharp peaks of rises and falls in the cytokine levels which lead to undesirable effects on the tissues including the pulp [16], whereas light and continuous forces maintain high cytokine levels, which are necessary for continuous periodontal remodeling, for a longer time [17].

Intrusion applied to the teeth during orthodontic treatment is thought to have the greatest impact on the apical region. Therefore, a significant reduction in the BF during the application of a continuous intrusive force is expected. This is substantiated by results from trauma studies where intrusion injuries cause the highest percentage of pulp necrosis particularly in teeth with closed apices, compared to other luxation injuries [18]. Brodin et al. [10] measured the effect of tooth intrusion and extrusion on BF of human incisors (6 subjects). They reported that extrusion of the teeth gave no significant changes in BF during loading or unloading, while intrusion of the teeth reduced the BF by 20% during the first minute after force application. Subsequently the BF gradually increased and returned to the pre-stimulus level 3 min after unloading.

On the other hand, Sabuncuoglu and Ersahan [19] showed that BF decreased in the incisor teeth when subjected to 3 days of either light (40 g) or heavy (120 g) orthodontic intrusion, and the BF values returned to their baseline levels after 3 weeks of either light (40 g) or heavy (120 g) intrusion. Similarly, they demonstrated a short-term regressive change in BF during continuous molar intrusion with mini-implants, which tended to return to baseline values by the end of the observation period (6 months) [15]. Furthermore, Wu et al. [20] reported that the use of larger dimension AW increased the pressure stresses on the periodontal ligament and the alveolar bone, which might add to the risk of root resorption and irreversible pulp changes.

In the current orthodontic practice, 0.17 × 0.025-inch stainless steel (SS), 0.019 × 0.025-inch (SS), and 0.021 × 0.025-inch β-titanium (TMA) AWs are used for COS leveling during orthodontic treatment [5]. The smaller AW/bracket-slot play when 0.021 × 0.025-inch TMA AW is used has the advantage of more torque control and a less low incisor proclination during leveling. However, the effect of using larger AW dimensions with high frictional forces on pulp tissues during orthodontic treatment is not known yet. Therefore, it was assumed that the type and size of the AW might have a different impact on the BF during COS leveling with intrusive and extrusive forces. The previously reported studies regarding BF changes during extrusion and intrusion orthodontic forces were performed on individual teeth (incisors or upper molars), and to date, there are no published studies evaluating BF during lower arch leveling with lower molar extrusion and lower incisor intrusion using different sized orthodontic AW. Therefore, to determine whether alterations in the BF of anterior and posterior teeth during arch leveling using continuous AW with reverse COS could affect the long-term vitality of teeth, this study was conducted with the following objectives:

1. To investigate BF changes of incisors subjected to orthodontic intrusive force during COS leveling using 0.017 × 0.025-inch stainless steel (SS) AW, 0.019 × 0.025-inch SS AW, and 0.021 × 0.025-inch β-titanium Titanium (TMA) AW at different time points (20 min, 48 h, 1 week, 1 month)
2. To investigate BF changes of molars and premolars subjected to orthodontic extrusive force during COS leveling using 0.017 × 0.025-inch SS AW, 0.019 × 0.025-inch SS AW, and 0.021 × 0.025-inch TMA AW at the above 4 time points
3. To compare BF changes based on tooth type and type of force (intrusive and extrusive forces) within the same AW size group
4. To compare BF changes between teeth in the three different AW size groups as per tooth type and type of force
5. To investigate the association between BF changes, type of force, AW type, tooth type, and the amount of change in COS

## Null hypothesis

There is no significant difference in BF changes between intrusive and extrusive forces during COS leveling regardless of the AW used: 0.017 × 0.025-inch SS AW, 0.019 × 0.025-inch SS AW, and 0.021 × 0.025-inch TMA AW.

## Material and methods

### Study design

This study was a randomized clinical trial with a 1:1:1 allocation ratio. The methods were not changed after trial initiation. The study was approved by the Institutional Review Board at the Jordan University of Science and Technology (approval number 78/117/2018). This trial was registered with ClinicalTrial.gov with identifier number NCT04549948.

The sample size was calculated using the G*power 3.1.9 program. Univariate analysis revealed significant variability between subjects (*F* = 4.45, *P* = 0.017, Partial Eta Squared = 0.15). Assuming a medium effect size difference (0.4) between groups, power analysis yielded a total sample size estimate of 14 subjects (7 patients per group) at a conventional alpha level (0.05) and desired power (1 – β) of 0.95. To build up for an attrition rate of 10%, initial recruitment targeted a total of 8 patients/group.

The participants for this study were recruited from patients attending postgraduate orthodontic clinics. All subjects who agreed to participate in the study signed a consent form for participation after clarifying the purpose of the intervention. Subjects were selected based on the inclusion criteria: age ≥ 16 years and ≤ 25 years, normally inclined or retroclined lower incisors, presence of deep bite, depth of COS ≥ 5 mm, non-extraction treatment plan, averaged or reduced lower vertical height, good oral hygiene, and healthy periodontium, and all permanent teeth are present except for the third molars.

Exclusion criteria were history of previous orthodontic treatment, teeth with root resorption, endodontically treated teeth, history of previous trauma, restoration on measured teeth, presence of a medical condition or being under medication that could affect the treatment, and smoking.

After recruiting patients who met the inclusion criteria and just before the insertion of the leveling AWs, the intervention was randomly allocated using the permuted random block size of 3 with a 1:1:1 allocation ratio by one research assistant (S.D.). The allocation sequence was concealed from the researcher (Y.N.) by sequentially numbered, opaque, sealed, and stapled envelopes before the intervention. Patients were then asked to pick a sealed envelope to assign the method of intervention. The methods were not changed after trial initiation. The patient was blinded to the intervention used, but it was not possible to blind the clinician during treatment. However, the measurements of the BF were performed by one research assistant (H.D.) who was blinded to the type of intervention used.

### Intervention

Thirty patients aged from 18 to 25 years who required fixed appliance orthodontic treatment were selected to participate in the study. All subjects were treated by the same orthodontic resident (Y.N.) using a pre-adjusted edgewise fixed appliance on upper and lower arches without extraction (American Orthodontics, 0.022 × 0.028-inch Roth prescription brackets). All AWs were ovoid in shape from 3 M Unetik company (Monrovia, California). A standardized bonding technique was applied according to the manufacturer’s instructions, and vertical bracket positioning was done using bracket gauge (4 mm from incisal tip for incisors, 4.5 mm from occlusal tip for canines and premolars).

Teeth alignment started with round 0.016-inch Nickel Titanium (NiTi) AW which included the upper and lower second molars, and then a sequence of 0.018-inch-NiTi and 0.016 × 0.022-inch-NiTi was inserted before 0.017 × 0.025-inch-NiTi AWs were reached. Appointment visits were the same for all patients during the intervention (every 4 weeks). After alignment and before the insertion of the reverse COS leveling Aws, an alginate impression for lower arch was taken for all patients at this time point (T0: before leveling).

Afterward, and based on their allocation group, 3 different leveling continuous AWs were inserted as follows:

- *Group 1*: Leveling of COS using 0.017 × 0.025-inch SS AW
- *Group 2*: Leveling of COS using 0.019 × 0.025-inch SS AW
- *Group 3*: Leveling of COS using 0.021 × 0.025-inch TMA AW

In the 3 studied groups, a 5 mm depth reversed COS was placed in the interventional AWs using tweed plier distal to lower canines. Measurement of the applied reverse COS in the AWs was done using digital caliper and inserted without a cinch-back. The anterior labial crown torque was removed from all AWs by holding the AWs mesial to the first premolars with a pair of tweed pliers and “twisting’’ the AWs to achieve a zero torque “flat” surface anteriorly. This was further checked by holding the tweed pliers at the anterior and posterior segments of the AWs and observing the lack of torque anteriorly. All teeth were included in the fixed orthodontic appliance including lower second molars. Patients were instructed to contact the clinic within 24 h if any bracket debonded. After 1 month, an alginate impression was taken for the lower arch, and the COS change was recorded. Utility wax was used to cover the lower arch brackets so as not to distort the impression upon removal from the mouth.

## Outcomes

### Primary outcome: blood flow (BF) (Fig. 1)

**Fig. 1:**
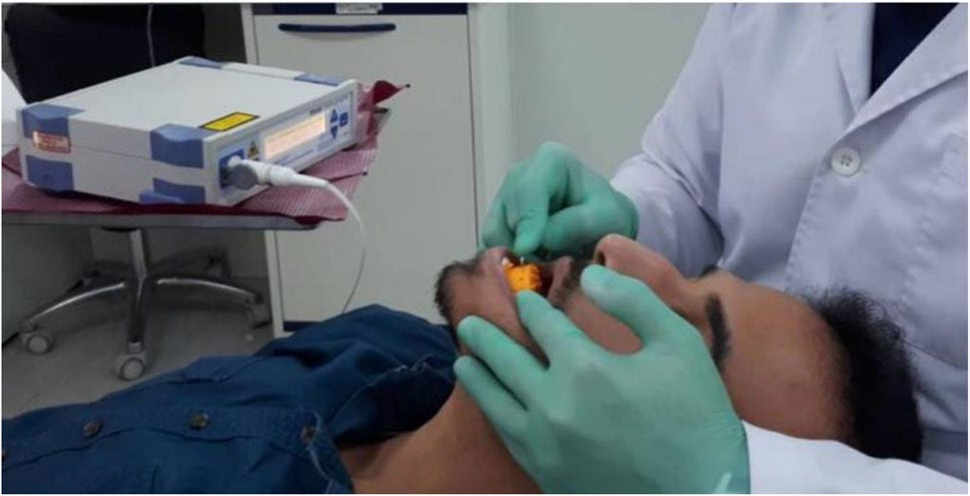
BF measurement using LDF (courtesy of Alhaija et al. 2021) [36]

Measurements of the BF were taken by the use of LDF (Moor lab, Moor instruments, UK) with a wavelength of 780 nm and a dental probe MP 13 (Moor instruments, UK; 2 fibers, 0.25 mm diameter, centers 0.5 mm spaced a part). The flowmeter was calibrated according to the manufacturer’s instructions. Room temperature was maintained from 20 to 25 °C. Volunteers were provided with 15 min’ rest before each session.

Before starting the measurements, a silicone splint was fabricated to stabilize the dental probe during the measurements. The retentive areas of the brackets were covered with a layer of utility wax. Holes were made below the imprints of the brackets in the mold with a stainless-steel drill of 1.5 mm diameter to allow the probe to pass through the mold to touch the teeth to allow measurement of teeth on the same position at different times. The silicone splints were fabricated to extend over the attached gingiva. The retentive areas of the brackets and the labial gingiva were covered with utility wax. In addition, 4 cotton rolls were applied in the gingival sulcus to keep the lower lip and cheeks away. This helped in isolating the teeth during measurements to minimize contamination of the blood flow signals from adjacent tissues [21].

BF was recorded at 5 points:

1. Before placement of interventional AWs in both groups. These values were considered the basal blood flow (T0).
2. Twenty minutes after placement of the interventional AW (T1).
3. Forty-eight hours after placement of the interventional AW (T2).
4. One week after placement of the interventional AW (T3).
5. One month after placement of the interventional AW (T4).

### Secondary outcome: depth of COS

The depth of COS was measured manually just before the placement of interventional AW and at the end of the intervention (1 month) using a digital caliper as the perpendicular distance between the deepest cusp tip and a flat plane that was laid on top of the mandibular dental cast, touching the incisal edges of the central incisors and the distal cusp tips of the second molars. It was measured on the right and left sides of the mandibular arch, and the average value was included in the analysis. All dental casts were trimmed and mounted equally on a dental surveyor to ensure accurate results.

### Method error

Measurement error using Dahlberg formula and Houston’s coefficient of reliability was calculated. Dahlberg error was 0.6 PU for BF and 0.1 mm for COS, and the coefficients of reliability were above 88% indicating substantial agreement.

### Statistical analysis

Data analysis was carried out using SPSS (28.0, SPSS Inc., NY, USA). Descriptive statistics for BF and COS depth at different time intervals were calculated. The Shapiro–Wilk test was applied to assess the normality of numeric data, and the result indicated that data were not normally distributed. The Wilcoxon signed-rank test was applied to detect differences between the right and left sides. The non-parametric Friedman test with a pairwise comparison of related samples and Bonferroni correction for multiple tests was applied to examine within-group differences in BF at the different time points. Kruskal–Wallis H test was used to detect differences between groups (AW groups and type of force subgroups). Linear regression analysis was applied to determine any association between BF changes within the first 20 min and the first 48 h of force application and AW size, type of force, tooth type, and the COS depth change. The *P*-value was set at 0.05 level.

## Results

**Fig. 2:**
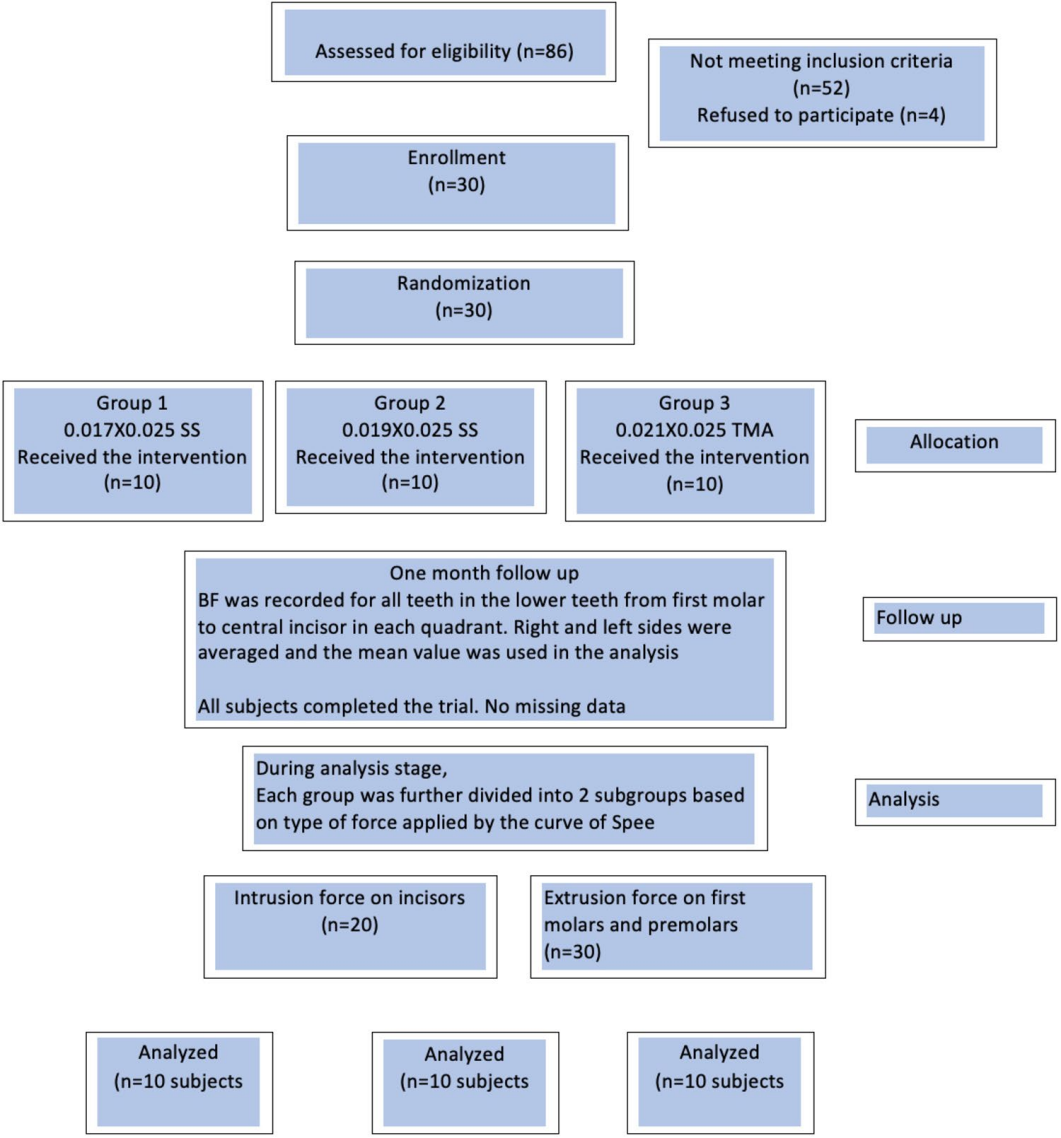
CONSORT flow chart showing patients’ flow during the trial

Thirty subjects (20 females and 10 males) received the planned intervention. Complete records for all subjects were available during the analysis stage. The age averaged 21.60 (3.62) years, 23.50 (4.66) years, and 20.20 (2.51) years in groups 1, 2, and 3, respectively. Before the intervention, the lower incisors’ inclination was 94.8 (3.7), 92.5 (4.0), and 93.4 (3.6) degrees, and the maxillary/mandibular plane angle was 24.1 (4.3), 24.6 (4.8), and 23.9 (2.9) degrees in groups 1, 2, and 3, respectively (*P* > 0.05). The participants’ flowchart is presented in Fig. 2. At T0, there were 30 subjects. None of the subjects were excluded from the analysis, and at the final analysis stage (T4), there was full data for 30 patients (10/group). Negative outcomes were not reported by any patients during the trial.

Teeth on both sides were assessed. The Wilcoxon signed-rank test revealed no significant differences in BF changes between the right and left sides within each group. Therefore, the right and left sides were averaged, and the mean BF values were used in the final analysis. The results are presented according to tooth type and type of applied force. The incisors were analyzed in the intrusion group (total of 60 teeth/20 teeth in each AW group), while the extrusion group was subdivided into 2 subgroups: premolars only (total of 60 teeth/20 teeth in each AW group) and first molars only as the second subgroup (total of 30 teeth/10 teeth in each AW group).

## Primary outcome

### Based on tooth type

The medians and the interquartile range (IQR) for the BF measurements per tooth type are shown in Table 1. At T0, the BF values ranged from 2.15 mm/s in central incisors to 3.09 mm/s in the first molars. At T1, it dropped and ranged from 1.74 mm/s in central incisors to 2.41 mm/s in the first molars. At T4, it ranged from 2.21 mm/s in central incisors to 3.48 mm/s in the lateral incisor tooth.

**Table 1:** The median and interquartile range (IQR) of the BF in the examined teeth within each group at the different time points

Tooth	T0 Median (IQR)	T1 Median (IQR)	T2 Median (IQR)	T3 Median (IQR)	T4 Median (IQR)
Group 1: 0.017 × 0.025 SS AW (*n* = 10 patients)					
First molar	3.01 (3.01–3.23)	2.41 (2.41–2.56)	2.74 (2.74–2.88)	2.89 (2.89–3.16)	3.18 (3.18–3.36)
Second premolar	2.60 (2.48–2.77)	2.29 (2.07–2.40)	2.58 (2.27–277)	2.61 (2.47–2.80)	2.78 (2.61–2.99)
First premolar	2.69 (2.28–2.86)	2.08 (2.01–2.39)	2.45 (2.18–2.65)	2.54 (2.50–2.73)	2.76 (2.28–3.01)
Canine	2.61 (2.19–2.96)	2.24 (1.97–2.45)	2.59 (2.39–2.71)	2.49 (2.36–2.56)	2.74 (2.67–2.89)
Lateral incisor	2.16 (2.09–2.40)	1.85 (1.68–2.18)	2.13 (1.60–2.30)	2.29 (2.10–2.53)	2.45 (2.13–2.60)
Central incisor	2.15 (1.97–2.38)	1.88 (1.68–2.01)	2.18 (1.77–2.20)	2.32 (1.99–2.49)	2.21 (1.97–2.46)
Group 2: 0.019 × 0.025 SS AW (*n* = 10 patients)					
First molar	2.68 (2.48–2.83)	2.14 (2.08–2.30)	2.51 (2.39–2.71)	2.55 (2.44–2.66)	2.99 2.93–3.18)
Second premolar	2.59 (2.45–2.79)	2.11 (2.08–2.22)	2.44 (1.98–2.76)	2.36 (2.14–2.65)	2.78 (2.64–2.91)
First premolar	2.58 (2.45–2.79)	2.20 (2.08–2.22)	2.40 (1.98–2.76)	2.64 (2.14–2.65)	2.76 (2.64–2.91)
Canine	2.49 (2.20–2.89)	2.05 (1.94–2.31)	2.40 (2.13–2.51)	2.54 (2.25–2.78)	2.66 2.37–2.86)
Lateral incisor	2.21 (1.96–2.39)	1.90 (1.49–2.04)	2.21 (1.99–2.41)	2.30 (2.15–2.58)	3.49 2.39–2.84)
Central incisor	2.28 (2.11–2.35)	1.74 (1.63–2.04)	2.21 (1.89–2.35)	2.23 (2.14–2.46)	2.60 (2.43–2.64)
Group 3: 0.021 × 0.025 TMA AW (*n* = 10 patients)					
First molar	3.09 (2.89–3.28)	2.40 (2.18–2.53)	2.48 (2.27–2.84)	2.73 (2.37–3.05)	3.00 (2.70–3.36)
Second premolar	2.54 (2.39–2.93)	2.06 (1.99–2.20)	2.25 (2.04–2.48)	2.53 2.06–2.71)	2.76 2.54–2.94)
First premolar	2.58 (2.39–2.93)	2.19 1.99–2.20)	2.30 (2.04–2.48)	2.29 (2.06–2.71)	2.78 (2.54–2.94)
Canine	2.44 (2.91–2.71)	2.15 (1.96–2.31)	2.20 (2.08–2.34)	2.34 (1.99–2.55)	2.51 (2.49–2.79)
Lateral incisor	2.25 (2.15–2.36)	1.96 (1.96–2.13)	2.15 (1.81–2.33)	2.15 (1.95–2.49)	2.40 (2.34–2.59)
Central incisor	2.23 (2.14–2.35)	1.80 (1.63–2.02)	2.16 (1.89–2.23)	2.15 (1.93–2.41)	2.30 (2.14–2.54)

*T0* at the baseline, *T1* 20 min after, *T2* 48 h after, *T3* one week after, and *T4* one month after the application of orthodontic force using the interventional archwires

^*^P < 0.05, **P < 0.01, ***P < 0.001

Comparisons of BF changes within and between the different AW groups according to type of tooth are presented in Table 2. In the 3 AWs group, BF of all measured teeth was reduced 20 min after force application. However, a statistically significant reduction was reached in the first molars and canines in group 1, in all teeth except the lateral incisors in group 2, and in all teeth except canines and lateral incisors in group 3. Afterwards, the BF values started to increase until the baseline values were almost restored within 1 week. Later, BF values increased more than their baseline values but did not reach any statistically significant level (*P* > 0.05).

**Table 2:** Medians, within group, and between group test statistics for the BF changes per tooth type at the different time points

	First molar		Second premolar		First premolar		Canine		Lateral incisors		Central incisors	
	Median	Standardized test statistics (*P* value adjusted)	Median	Standardized test statistics (*P* value adjusted)	Median	Standardized test statistics (*P* value adjusted)	Median	Standardized test statistics (*P* value adjusted)	Median	Standardized test statistics (*P* value adjusted)	Median	Standardized test statistics (*P* value adjusted)
Within + group standardized test statistics and adjusted *P* value												
Group 1: 0.017 × 0.025 SS AW (*n* = 10 patients)												
T0-T20	− 060	− 3.68 (0.002)***	− 0.48	− 2.76 (0.058)	− 0.45	− 2.76 (0.058)	− 0.30	− 3.11 (0.019)*	− 0.34	− 2.33 (0.196)	− 0.23	− 2.33 (0.196)
T20-T48	0.33	− 1.41 (1.000)	− 0.26	− 1.84 (0.660)	0.33	− 1.91 (0.562)	0.25	− 2.48 (0.133)	0.18	− 1.06 (1.000)	0.21	− 1.77 (0.771)
T48-T1W	0.21	− 0.71 (1.000)	0.05	− 1.06 (1.000)	0.05	− 0.35 (1.000)	− 0.11	− 1.13 (1.000)	0.31	− 1.63 (1.000)	0.19	− 1.13 (1.000)
T1W-T1M	0.35	− 2.69 (0.072)	0.19	− 0.29 (1.000)	0.24	− 0.71 (1.000)	− 0.15	− 1.69 (0.072)	0.21	− 1.13 (1.000)	0.08	− 0.35 (1.000)
Group 2: 0.019 × 0.025 SS AW (*n* = 10 patients)												
T0-T20	− 0.48	− 3.39 (0.007)**	− 0.58	− 3.32 (0.009)**	− 0.40	− 3.04 (0.024)*	− 0.60	− 3.54 (0.004)**	− 0.31	− 1.77 (0.771)	− 0.40	− 3.04 (0.024)*
T20-T48	0.33	2.19 (0.284)	0.27	− 1.49 (1.000)	0.15	− 1.70 (0.897)	0.29	− 1.70 (897)	0.38	− 2.33 (0.196)	0.22	− 2.48 (0.133)
T48-T1W	0.09	0.07 (0.944)	0.15	− 0.07 (1.000)	0.14	− 0.28 (1.000)	0.20	− 1.34 (1.000)	0.15	− 1.13 (1.000)	0.16	− 0.71 (1.000)
T1W-T1M	0.42	2.62 (0.089)	0.31	− 2.69 (0.072)	0.16	− 2.33 (0.196)	0.08	− 1.71 (1.000)	0.24	− 1.34 (1.000)	0.26	− 1.91 (0.562)
Group 3: 0.021 × 0.025 TMA AW (*n* = 10 patients)												
T0-T20	− 0.75	− 4.03 (0.001)***	− 0.53	− 3.39 (0.007)**	− 0.45	− 2.97 (0.030)*	− 0.34	− 2.76 (0.058)	− 0.21	− 1.91 (0.562)	− 0.41	− 3.68 (0.002)**
T20-T48	0.15	− 0.99 (1.000)	0.15	− 1.41 (1.000)	0.30	− 1.41 (1.000)	0.01	− 0.35 (1.000)	0.15	− 0.85 (1.000)	0.28	− 2.55 (0.109)
T48-T1W	0.29	− 1.56 (1.000)	0.15	− 0.92 (1.000)	0.03	− 0.14 (1.000)	0.13	− 1.20 (1.000)	0.04	− 0.64 (1.000)	0.09	− 0.21 (1.000)
T1W-T1M	0.34	− 1.91 (0.0562)	0.35	− 2.19 (0.284)	0.30	− 2.40 (0.162)	0.28	− 2.26 (0.237)	0.19	− 2.40 (0.162)	0.04	− 1.70 (0.897)
Between groups Kruskal–Wallis Test Statistics												
T0-T20	2.83 (0.243)		2.05 (0.358)		0.01 (0.998)		1.42 (0.491)		0.71 (0.701)		1.99 (0.370)	
T20-T48	2.35 (0.309)		0.96 (0.620)		0.42 (0.812)		8.00 (0.018)*		5.15 (0.076)		2.86 (0.240)	
T48-T1W	0.56 (0.755)		0.29 (0.864)		0.34 (0.844)		3.18 (0.204)		1.34 (0.511)		1.25 (0.536)	
T1W-T1M	3.75 (0.154)		3.04 (0.219)		1.68 (0.433)		2.81 (0.245)		1.16 (0.561)		2.33 (0.312)	

*T0* At the baseline, *T1* 20 min after, *T2* 48 h after, *T3* 1 week after, and *T4* 1 month after the application of orthodontic force using the interventional archwires

^*^P < 0.05, **P < 0.01, ***P < 0.001

Similar BF changes were observed after the application of orthodontic force using different AW sizes and materials except for the lower canines, where it showed more reduction (− 3.11 mm/s and − 2.48 mm/s in groups 1 and 2, respectively) and more recovery (1.13 mm/s and 1.69 mm/s) in SS groups compared to the TMA group (− 2.76 mm/s and 0.35 mm/s) (*P* < 0.05).

### Based on force type

The medians and the IQR for the BF measurements per type of force applied are shown in Table 3. At T0, the BF values ranged from 2.23 mm/s in the intrusion group to 3.01 in the extrusion group. At T1, the BF values dropped and ranged from 1.80 mm/s in the intrusion group to 2.41 mm/s in the extrusion group. At T4, it increased and ranged from 2.35 mm/s in the intrusion group to 3.18 mm/s in the extrusion group.

**Table 3:** Medians and interquartile range (IQR) of BF in teeth subject to intrusive and extrusive orthodontic forces and Friedman test results to detect within-group differences at the different time points

Subgroups	Tooth	T0 Median (IQR)	T1 Median (IQR)	T2 Median (IQR)	T3 Median (IQR)	T4 Median (IQR)	Within group Chi square	*P* value
Group 1: 0.017 × 0.025 SS AW (*n* = 10 patients)								
Extrusion	First molar (*n* = 10)	3.01 (3.01–3.23)	2.41 (2.41–2.56)	2.74 (2.74–2.88)	2.89 (2.89–3.16)	3.18 (3.18–3.36)	28.48	*P* < 0.001***
	Second premolar (*n* = 10) First premolar (*n* = 10)	2.65 (2.48–2.79)	2.21 (2.05–2.39)	2.50 (2.26–2.71)	2.57 (2.50–2.76)	2.76 (2.52–2.99)	27.53	*P* < 0.001***
Intrusion	Lateral incisor (10)	2.26 (2.05–2.37)	1.86 (1.70–2.11)	2.15 (1.71–2.22)	2.30 (2.10–2.50)	2.35 (2.12–2.56)	30.14	*P* < 0.001***
	Central incisor (10)							
Group 2: 0.019 × 0.025 SS AW (*n* = 10 patients)								
Extrusion	First molar (*n* = 10)	2.68 (2.48–2.83)	2.14 (2.08–2.30)	2.51 (2.39–2.71)	2.55 (2.44–2.66)	2.99 2.93–3.18)	25.96	*P* < 0.001***
	Second premolar (*n* = 10) First premolar (*n* = 10)	2.59 (2.39–2.79)	2.13 (1.98–2.26)	2.41 (2.12–2.81)	2.46 (2.18–2.74)	2.78 2.61–2.91)	39.59	*P* < 0.001***
Intrusion	Lateral incisor (*n* = 10) Central incisor (*n* = 10)	2.23 (2.09–2.35)	1.80 (1.63–2.02)	2.21 (1.96–2.35)	2.26 (2.16–2.49)	2.55 (2.43–2.69)	52.17	*P* < 0.001***
Group 3: 0.021 × 0.025 TMAAW (*n* = 10 patients)								
Extrusion	First molar (*n* = 10)	3.09 (2.89–3.28)	2.40 (2.18–2.53)	2.48 (2.27–2.84)	2.73 (2.37–3.05)	3.00 (2.70–3.36)	29.45	*P* < 0.001***
	Second premolar (10) First premolar (10)	2.58 (2.41–2.89)	2.10 (2.00–2.23)	2.30 2.08–2.45)	2.52 (2.05–2.69)	2.75 2.51–2.92)	42.92	*P* < 0.001***
Intrusion	Lateral incisor (10) Central incisor (10)	2.23 (2.18–2.34)	1.91 (1.69–2.09)	2.16 (1.88–2.32)	2.15 (1.98–2.42)	2.36 (2.16–2.58)	37.51	*P* < 0.001***

*T0* at the baseline, *T1* 20 min after, *T2* 48 h after, *T3* 1 week after, and *T4* 1 month after the application of orthodontic force using the interventional archwires

^***^P < 0.001

Comparisons of BF changes within and between the different AW groups according to type of applied force are presented in Table 4.

**Table 4:** Medians, within-group test statistics, and *P* values, between the intrusive and extrusive subgroup test statistics and *P* values, and between the 3 AW group test statistics and *P* values

Variable	Extrusive forces				Intrusive forces		
	Premolars (*n* = 20)		First molar (*n* = 10)		Incisors (*n* = 20)		Kruskal–Wallis test statistics (*P* value) between force-type subgroups
	Median	Test statistics (*P* value)	Median	Test statistics (*P* value)	Median	Test statistics (*P* value)	
Group 1: 0.017 × 0.025 SS AW (*n* = 10 patients)							
T0-T20	− 0.48	− 3.90 (0.001)***	− 0.60	− 3.68 (0.002)***	− 0.29	− 3.30 (0.010)**	7.06 (0.029)*
T20-T48	0.26	− 2.65 (0.080)	0.33	− 1.41 (1.000)	0.19	− 2.00 (0.455)	4.64 (0.098)
T48-T1W	0.05	− 1.00 (1.000)	0.21	− 0.71 (1.000)	0.21	− 1.95 (0.512)	1.16 (0.560)
T1W-T1M	0.22	− 1.15 (1.000)	0.35	− 2.69 (0.072)	0.18	− 1.05 (1.000)	5.14 (0.076)
Group 2: 0.019 × 0.025 SS AW (*n* = 10 patients)							
T0-T20	− 0.50	− 4.50 (0.000)***	− 0.48	− 3.39 (0.007)**	− 0.40	− 3.40 (0.007)**	0.63 (0.729)
T20-T48	0.24	− 2.25 (0.244)	0.33	2.19 (0.284)	0.35	− 3.40 (0.007)**	2.50 (0.287)
T48-T1W	0.14	− 0.150 (1.000)	0.09	0.07 (0.944)	0.15	− 1.30 (1.000)	0.09 (0.958)
T1W-T1M	0.19	− 3.55 (0.004)**	0.42	2.62 (0.089)	0.25	− 2.30 (0.214)	4.17 (0.124)
Group 3: 0.021 × 0.025 TMAAW (*n* = 10 patients)							
T0-T20	− 0.48	− 4.50 (0.001)***	− 0.75	− 4.03 (0.001)***	− 0.30	− 3.95 (0.001)***	8.09 (0.018)*
T20-T48	0.19	− 2.00 (0.455)	0.15	− 0.99 (1.000)	0.21	− 2.40 (0.164)	0.43 (0.807)
T48-T1W	0.05	− 0.75 (1.000)	0.29	− 1.56 (1.000)	0.05	− 0.60 (1.000)	1.72 (0.423)
T1W-T1M	0.35	− 3.25 (0.012)*	0.34	− 1.91 (0.0562)	0.15	− 2.90 (0.037)*	4.17 (0.124)
Kruskal–Wallis test statistics (*P* value) between the 3 AW groups							
	Extrusive forces Premolars (*n* = 20)		Extrusive forces First molar (*n* = 10)		Intrusive forces Incisors (*n* = 20)		
T0-T20	0.97 (0.615)		2.83(0.243)		0.76 (0.685)		
T20-T48	8.27 (0.016)*		2.35 (0.309)		1.08 (0.583)		
T48-T1W	2.59 (0.274)		0.56 (0.755)		0.24 (0.886)		
T1W-T1M	3.46 (0.178)		3.75 (0.154)		3.79 (0.150)		

*T0* at the baseline, *T1* 20 min after, *T2* 48 h after, *T3* 1 week after, and *T4* 1 month after the application of orthodontic force using the interventional archwires

^*^P < 0.05, **P < 0.01, ***P < 0.001

Within the same AW group at the different time intervals, similar BF changes were found within the first 20 min of intrusion and extrusion force application. In group 1, BF values were restored within 1 week, whereas it continued to increase in the 0.019 × 0.025-inch SS group (premolar extrusion subgroup), and in the TMA group (premolar extrusion and the incisor intrusion subgroups).

Within the same AW groups, when the extrusion and intrusion subgroups were compared, significant differences in BF changes were observed in groups 1 and 3 during the first 20 min of force application (*P* < 0.05), while in group 2, no statistically significant differences were detected at any time point (Table 4).

Similar BF changes were recorded in teeth subjected to extrusion and intrusion forces using the 3 different AWs. However, in the premolars’ extrusion group, significant BF increase toward baseline values was observed within the first 48 h of force application in the SS AW groups (*P* = 0.016).

Regression analysis results (Table 5) indicated that BF changes within the first 20 min of force application were significantly associated with tooth type (*P* = 0.001) and the amount of COS depth change (*P* < 0.001). However, none of these factors showed any association with BF changes at subsequent time points (*P* > 0.05).

**Table 5:** Results of regression analysis to predict BF changes within the first 20 min and 48 h of orthodontic force application

	Unstandardized coefficient B (SE)	Standardized coefficient Beta	*T* value	*P* value	95% confidence interval for B
T20-T0 (*R* = 0.289, *F* = 4.43, *P* = 0.005)					
AW	− 0.05 (0.03)	− 0.13	− 1.88	0.062	− 0.11–0.003
Type of orthodontic force	− 0.02 (0.04)	− 0.04	− 0.45	0.657	− 0.09–0.06
Tooth type	− 0.05 (0.02)	− 0.27	− 3.36	0.001***	− 0.08 to − 0.02
Amount of change in the CoS/month	− 0.21 (0.05)	− 0.29	− 3.36	< 0.001***	− 0.30 to − 0.11
T48-T20 (*R* = 0.09, *F* = 0.41, *P* = 0.745)					
AW	− 0.03 (0.03)	− 0.09	− 1.21	0.228	− 0.09–0.02
Type of orthodontic force	0.03 (0.04)	0.07	0.87	0.388	− 0.04–0.10
Tooth type	0.01 (0.01)	0.05	0.61	0.543	− 0.02–0.04
Amount of change in the CoS/month	0.07 (0.05)	0.11	1.46	0.147	− 0.03–0.17

AW were coded as 1: 0.017 × 0.025SS, 2: 0.019 × 0.025 SS, 3: 0.021 × 0.025 TMA; type orthodontic forces were coded as 1: extrusion, 2: intrusion and tooth type by its number 1: central incisor – 6: first molar

## Secondary outcome

### Reduction of COS

Within the 1-month trial, COS was reduced in all groups. The baseline depth of the COS before the intervention averaged 5.30±0.46 mm, 5.60 ± 0.92 mm, and 5.40±0.49 mm in groups 1, 2, and 3, respectively. After 1 month, COS was on average 4.40±0.49 mm, 4.70±0.91, and 4.70±0.46 in groups 1, 2, and 3, respectively (*P* > 0.05). The reduction of COS was significant in all AW sizes’ groups (*P* < 0.001).

## Discussion

Although BF changes during extrusion and intrusion orthodontic forces have been previously reported [10, 15, 19, 22–25], these studies were done on individual teeth (incisors or upper molars) with forces applied in a different way to that during conventional fixed orthodontic treatment. Since it has been shown that during orthodontic treatment, COS is leveled by molars and premolars extrusion, and lower incisors intrusion [2–5], the current study was conducted to investigate and compare BF in the lower teeth during lower arch COS leveling using different AWs. The null hypothesis of this study was partly rejected as there were significant differences in the BF changes between the intrusion and extrusion forces using different AW size and material.

In the current orthodontic practice, 0.017 × 0.025-inch SS, 0.019 × 0.025-inch SS, and 0.21 × 0.025 TMA AWs are used for COS leveling during orthodontic treatment. The reported stiffness values for 0.019 × 0.025-inch SS AW are higher than 0.021 × 0.025-inch-TMA AW which means the amount of the delivered force when using 0.19 × 0.025-inch SS is higher [26]. On the other hand, while TMA AW generates gentle forces and delivers approximately half the force of SS AW [27], 0.021 × 0.025-inch TMA AW has less AW/bracket-slot play (due to increased AW dimension) and has higher frictional resistance compared to SS AWs [28]. The smaller AW/bracket-slot play when 0.021 × 0.025-inch TMA AW is used has the advantage of more torque control and a less lower incisor proclination during leveling. However, the effect of using larger AW dimension with high frictional forces on pulp tissues during orthodontic treatment is not known yet. Furthermore, Wu et al. [20] reported that the use of larger dimension AWs increased the pressure stresses on the PDL and alveolar bone which might add the risk of root resorption and irreversible pulp changes. Therefore, it was assumed that the type and size of the AW might have different impact on the BF during COS leveling with intrusive and extrusive forces.

In the current study, only subjects with good oral hygiene and healthy periodontium were included. The oral health status during the study was maintained by giving the patients oral hygiene instructions both verbally and using social media [29]. Plaque accumulation is a major concern during orthodontic treatment, where brackets, AWs, and elastics hinder access to good oral hygiene measures causing worsening of the oral health status [30]. However, no evidence of any significant difference in oral hygiene levels among the different orthodontic appliances was reported [31].

It has been demonstrated that tooth morphology affects the distribution, the amount of orthodontic force, and the developed strain within the PDL [32]. Therefore, the extrusion force was studied in the first molars and premolars separately.

Different types of tooth movement were reported during leveling the COS in subjects with different vertical proportions. Rozzi et al. [33] demonstrated that in low-angle subjects, leveling of the COS occurs through buccal movement and intrusion of the mandibular incisors; in high-angle subjects, it occurs through extrusion and uprighting of the posterior teeth. In the current study, all included subjects had average or reduced vertical proportions to ensure that arch leveling will be similarly produced among subjects. In the present study, there was a decrease in BF 20 min after the placement of the interventional AWs irrespective of their size, tooth type, or type of force application. The reduction in BF is attributed to the force applied to the vessels that enter and exit the apical foramen, which get constricted. The resulting vascular compression thus creates a reduction in the BF [34]. Extrusion forces cause stretching of the pulpal vasculature, and if severe may result in its rupture, while intrusive forces cause compression and may collapse depending on the magnitude of force. The BF began to recover after 48 h of force application and returned to its baseline levels after 1 week, which indicates that the decaying of the forces had begun, and the inflammatory process of the initial phase was in the reverse.

Within the first 20 min of force application, BF showed more reduction in the extrusion subgroup as compared to the intrusion subgroup. Although intrusion is associated with more crushing of cells and vessels in the apical tissues than luxation injuries, and more detrimental effect on the vasculature of the pulp [18], this effect might have been superseded by higher trauma to the apical vasculature by virtue of larger vertical movements during extrusion. Nasrawi et al. [5] assessed the amount of lower intrusion and molar extrusion during COS leveling using 3 different AWs. They reported that COS was leveled by 0.41 mm, 0.06 mm, and 0.16 mm of lower molar extrusion and by 0.04 mm, 0.24 mm, and 0.58 mm of lower incisor intrusion when 0.017 × 0.025 SS AW, 0.019 × 0.025 SS AW, and 0.021 × 0.025 TMA AW were used, respectively [18].

In the current study, significant BF recovery occurred within 48 h of force application (intrusion and extrusion) in group 1, while it continued to increase in the premolar extrusion subgroups in 0.019 × 0.25-inch SS and 0.21 × 0.025-inch TMA AWs and in the intrusion subgroup in the TMA AW group. The continued BF increase after 1 week of extrusive force application in groups 2 and 3 could be explained by the higher force applied to the teeth using the larger dimension AWs in groups 2 and 3. Also, the less AW/bracket play in group 3 may have produced more frictional forces.

These results agree with previous studies [22, 23, 35] and in partial agreement with Brodin et al. [10], who reported that extrusion of the teeth gave no significant changes in BF, while intrusion reduced the BF by 20% during the first minute after force application, and BF returned to the pre-stimulus level 3 min after unloading. Variation in results may be attributed to the type of tooth being investigated and to the amount of vertical movement induced during either intrusion or extrusion. Contrary to the finding of the current study, Barwick and Ramsay [24] reported that despite the application of heavy intrusive forces for a short time, there was no alteration in the BF in human maxillary central incisors. The small sample size, type of teeth tested, and the size of their apical foramen, force application methods, duration of force application, and the different methodology used to assess BF may explain the variation in results.

These results were also inconsistent with studies that used NiTi AWs; McDonald and Pitt Ford [25] reported an increase in BF at 24 h and 48 h using a removable appliance, while other studies [22, 33, 35, 36] reported a maximum BF reduction 72 h after orthodontic force application and restored BF to original values after 1 month. NiTi AWs are more flexible as they have a lower modulus of elasticity compared to stainless steel wires, with better mechanical properties that allow them to deliver lower continuous force over longer periods. The use of different time points in the current study, different AW sizes and materials, and different force levels in the previous studies explains the difference in the reported findings.

When comparing BF changes between the 3 AWs, only the canines showed significant differences. The canines showed more BF changes (more BF reduction during the first 20 min and more recovery during the first 48 h) when the SS AWs were used. This may be due to higher forces applied to the canines when using the stiffer SS AWs. Also, the position of the canines in the middle of the lower arch connecting the intrusion and extrusion parts of the reverse COS AWS may have subjected the canines to more complex types of orthodontic forces.

When the type of force was compared between the 3 AWs, premolars subgroup showed more BF return toward their baseline value in group 2 (0.019 × 0.025-inch SS AW) as compared to group 3 (TMA AW) within the first 48 h of force application. As the premolars are in the deepest point of the accentuated COS, they are subjected to more extrusive forces when COS is placed in the AW. This indicates that the premolars in SS group may have been subjected to more insults due to higher forces that necessitated an increase in BF changes to help in recovery.

In the current study, 1 month after force application, BF increased beyond its original value. This was contrary to the previous studies [15, 22, 33, 35, 36]. Abu Alhaija et al. [22, 23, 35, 36] reported no difference in the BF 1 month after orthodontic force application and the baseline values using NiTi AW for orthodontic alignment. Also, Sabuncuoglu and Ersahan [15] demonstrated that BF values tend to return to their baseline levels after 3 weeks in both light (40 g) and heavy (120 g) intrusive force groups. However, in their study [15], the intrusive force was applied on upper incisors using NiTi coil spring from 0.016 × 0.022-inch AW to a mini-implant, whereas in the current study, heavier and larger dimensions AWs were used for the COS leveling. The continued increase in BF in the current study even at 1 month may be explained by the fact that forces were higher and more damage has occurred to the pulp; therefore, in an attempt of the pulp to heal itself, hyperemia continued. This is a natural tendency of the pulp tissue to adapt to the aggression caused by force application. The inflammatory process will bring in the inflammatory cells and vasodilation, thus promoting tissue repair and generating new blood vessels [37]. Longer follow-up from 3 or 6 months may have shown more details on the recovery potential of the dental pulp in both types of forces (intrusion, extrusion).

Regression analysis revealed an association between BF changes with the type of tooth and the amount of COS depth changes. The change of the COS reflects the outcome of orthodontic force application. Therefore, it is expected to detect changes in the pulp as a result of this force. Based on the above results, it seems prudent to evaluate the amount of change in COS required and to select the type of AW that may produce the least amount of force to avoid detrimental pulpal effects during extrusion.

Limitations of the current study include the following: BF measurements were carried out during active orthodontic treatment, and teeth were not in a fixed position, the extrusive and intrusive forces were applied to teeth with different morphology, and the presence of the orthodontic appliance limits the measurement area and hinders the placement of a black rubber dam sheet during BF measurements to reduce signals from the gingival blood vessels [38, 39].

## Conclusions

- During CoS leveling, BF was reduced after 20 min and then started to increase at 48 h. It returned to its baseline values 1 week after AW insertion in all AW size groups and type of force subgroups.
- Similar BF changes were recorded in all teeth using different AWs sizes and materials.
- In groups 1 and 3, more BF reduction were found in the extruded premolars compared to that of the intruded incisors during the first 20 min of AW placement.
- After 1 week of reverse CoS AW placement, BF continued to increase in the extruded premolars in groups 2 and 3 and in the intruded incisors in group 3.
- In the premolars “extrusion subgroup,” BF showed more increase toward baseline values within the first 48 h of force application in the SS AW groups (groups 1 and 2).
- Tooth type and the amount of COS depth change were associated with BF changes within the first 20 min of force application.

## Generalizability

This is a single center study; therefore, generalizability of the results cannot be made.

## Data Availability

The data that support the findings of this study are available from the corresponding author upon reasonable request.
